# Clinical Significance of Elevated Levels of Soluble‐Form Immune Checkpoint Molecules in Patients With Aggressive Adult T‐Cell Leukemia‐Lymphoma

**DOI:** 10.1002/jha2.70046

**Published:** 2025-06-26

**Authors:** Shigeo Fuji, Junya Makiyama, Kuniko Takano, Ilseung Choi, Takeo Suzuki, Chihiro Suminaka, Mao Kuroishi, Yosuke Iwasaki, Hidenori Kasahara, Yuma Tada, Yasuhiro Shingai, Sayako Yuda, Takafumi Yokota, Jun Ishikawa

**Affiliations:** ^1^ Department of Hematology Osaka International Cancer Institute Osaka Japan; ^2^ Department of Hematology Sasebo City General Hospital Nagasaki Japan; ^3^ Department of Medical Oncology & Hematology Faculty of Medicine Oita University Oita Japan; ^4^ Department of Hematology Kyushu Cancer Center Fukuoka Japan; ^5^ Department of Medical & Scientific Affairs, Sysmex Corporation Kobe Japan; ^6^ Central Research Laboratories Sysmex Corporation Kobe Japan

**Keywords:** adult T‐cell leukemia‐lymphoma, immune checkpoint, sIL‐2R

## Abstract

**Background:**

Immune checkpoint (IC) pathways, including programmed death protein 1 (PD‐1), its ligand PD‐L1, and CTLA‐4, mediate negative regulatory signals in immune responses. Recent studies in autoimmune diseases and malignancies reported that the presence of the soluble form of these ICs would reflect the overall immune status. We assessed the clinical significance of these soluble‐form ICs in HTLV‐1 carriers and adult T‐cell leukemia‐lymphoma (ATL) patients.

**Methods:**

After obtaining informed consent, plasma from HTLV‐1 carriers and ATL patients was prospectively collected in participating centers. Plasma concentrations of soluble‐form PD‐1, PD‐L1, and CTLA‐4 (sPD‐1, sPD‐L1, and sCTLA‐4) were measured.

**Results:**

Ninety‐six cases were included (HTLV‐1 carriers, *n* = 4; indolent ATL, *n* = 14; aggressive ATL, *n* = 78). The median age at sampling was 65.5 (range, 23–85) years. Soluble factors levels were significantly higher in aggressive ATL than in indolent ATL (sPD‐1, *p* = 0.003; sPD‐L1, *p* = 0.017; sCTLA‐4, *p* < 0.001). These markers were strongly correlated with soluble IL‐2R (sPD‐1, *r* = 0.80; sPD‐L1, *r* = 0.61; sCTLA‐4, *r* = 0.82). Elevated soluble IC levels were significant adverse prognostic factors.

**Conclusion:**

Soluble IC levels were significantly higher in aggressive ATL than in indolent ATL, and predicted an adverse prognosis. Their clinical significance should be investigated in larger studies.

## 1 Introduction

The immune checkpoint (IC) pathways, including programmed death protein 1 (PD‐1), its ligand Programmed cell Death ligand 1 (PD‐L1), and Cytotoxic T‐lymphocyte antigen‐4 (CTLA‐4), have been shown to mediate negative regulatory signals that effectively inhibit the proliferation and function of T cells and impair antitumor immune responses [1, 2]. The immunosuppressive microenvironment provided by negative regulatory signaling pathways is an important component of tumor escape from the attack by the immune system in cancer patients [2]. Anti‐PD‐1 and PD‐L1 antibodies are used to treat various types of solid tumors and some hematological malignancies.

PD‐1 is mainly expressed on T cells, but is also presented on other immune cells such as natural killer (NK) cells, monocytes, dendritic cells (DCs), B cells, and regulatory T cells (Tregs) [3]. PD‐L1 is mainly expressed on tumor cells, but is also presented on other cells such as endothelial and epithelial cells [3]. Previous studies have reported the expression of PD‐1, PD‐L1, and CTLA‐4 on ATL cells and in microenvironements [4, 5, 6]. The presence of these immune checkpoint molecules was reported to be associated with a poor prognosis in patients with ATL [4, 5, 6].

In terms of IC, recent studies in autoimmune diseases and malignancies reported that the soluble forms of these ICs were detected in peripheral blood and suggested that the levels of these soluble factors would reflect the overall immune status in the body [7, 8, 9]. Furthermore, soluble‐form ICs also function as immune modulators [10, 11]. For instance, soluble PD‐L1 has been reported to induce apoptosis of normal T cells and impair their function [7]. Several studies reported the elevation of these soluble ICs in peripheral blood in patients with malignant lymphoma (e.g., diffuse large B‐cell lymphoma, Hodgkin lymphoma and NK/T cell lymphoma) [12, 13, 14, 15, 16, 17, 18, 19]. However, the biological activities and clinical significance of these soluble ICs remain undetermined in patients with ATL.

We aimed to evaluate the levels of the soluble form of these ICs in human T‐cell leukemia virus type I (HTLV‐1) carriers and ATL patients and analyze the correlation of these markers with other parameters and clinical subtypes of ATL, and the impact of these markers on the clinical outcomes of patients with ATL.

### 1.1 Patient and Methods

#### 1.1.1 Study Patients and Measurement of Samples

Plasma from HTLV‐1 carriers and ATL patients was prospectively collected in participating centers after obtaining informed consent from August 2017 to February 2023. Plasma concentrations of sPD‐1, sPD‐L1 and sCTLA‐4 were measured by an automated immunoassay system (Automated Immunoassay System HISCL‐5000; Notification Number: 28B1×10014000011, Sysmex Corporation) based on chemiluminescence magnetic technology using our own reagents (research use only, not commercially available). The HISCL system is highly sensitive, reproducible, and precise for quantitative determination of the concentrations of these soluble immune markers in human plasma, as previously described [20]. The plasma concentration of soluble interleukein‐2 receptor (sIL‐2R) was measured by an enzyme immunoassay. In this current study, we excluded patients with active malignancies other than ATL at the time of sampling. Aggressive ATL was defined as acute type, lymphoma type, or unfavorable chronic type; indolent ATL was defined as smoldering type or favorable chronic type [21]. The definition of unfavorable chronic type was as previously reported: elevated serum lactic dehydrogenase (LDH), elevated blood urea nitrogen (BUN), and decreased serum albumin levels [21].

### 1.2 Statistical Analysis

The primary endpoint in this study was the association between the plasma levels of soluble immune checkpoint molecules and the clinical subtype of ATL. The secondary endpoints included the association between the plasma levels of soluble immune checkpoint molecules and the sIL‐2R level, and overall survival (OS). OS was defined as the time from enrollment to death from any cause. Continuous variables are expressed as medians and interquartile ranges. Categorical variables are expressed as counts and percentages. Patient and disease characteristics were compared using the Mann–Whitney U test for continuous variables and the chi‐square test for categorical variables. The probability of OS was calculated using the Kaplan–Meier method, and differences between groups were assessed using the log‐rank test. We excluded HTLV‐1 carriers from the survival analysis. A Cox proportional hazards regression model was used to analyze OS. For all analyses, two‐sided *p* values of < 0.05 were considered statistically significant. The cutoff thresholds used in each of the soluble ICs were the median values. All statistical analyses were performed with EZR (Jichi Medical University Saitama Medical Center, Saitama, Japan; http://www.jichi.ac.jp/saitama‐sct/ SaitamaHP.files/statmedEN.html), a graphical user interface for R version 1.54 (The R Foundation for Statistical Computing, Vienna, Austria) [22]. More precisely, it is a modified version of R commander designed to add statistical functions frequently used in biostatistics. Some statistical analyses were performed using the JMP software program (JMP Statistical Discovery LLC., USA). Local ethics committee approval was obtained (Osaka International Cancer Institute, No. 23103), and the study was conducted in accordance with the Declaration of Helsinki.

## 2 Results

**FIGURE 1:**
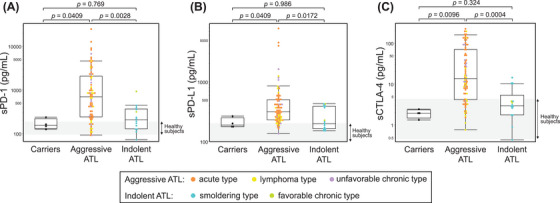
Discrimination performance of each immune checkpoint molecule for aggressive ATL. (A) sPD‐1, (B) sPD‐L1, (C) sCTLA‐4. Each gray area in the figure represents the interquartile range of healthy subjects (*n* = 50) from Goto *et al.* [20].

**FIGURE 2:**
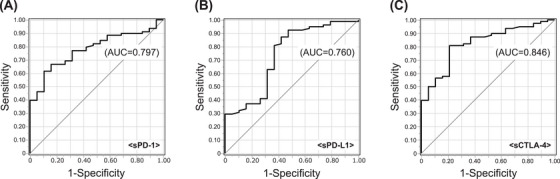
Discrimination performance of each immune checkpoint molecule for aggressive ATL, as determined by an analysis of ROC curve. (A) sPD‐1, (B) sPD‐L1, (C) sCTLA‐4.

The patient characteristics are summarized in Tables 1 and 2. A total of 96 cases were included (HTLV‐1 carriers, *n* = 4; smoldering type, *n* = 12; favorable chronic type, *n* = 2; unfavorable chronic type, *n* = 11; lymphoma type, *n* = 18; acute type, *n* = 49; Table 1). The median age at sampling was 65.5 (range, 23–85) years (Table 2). The median levels of sPD‐1, sPD‐L1, sCTLA‐4, and sIL‐2R were 454 pg/mL, 300 pg/mL, 10.1 pg/mL, and 3451 U/mL, respectively. The levels of soluble factors were significantly higher in aggressive ATL than in indolent ATL (sPD‐1, *p* = 0.003; sPD‐L1, *p* = 0.017; sCTLA‐4, *p* < 0.001; Figure 1A–C). The discrimination performance of each immune checkpoint molecule for aggressive ATL was determined by an ROC curve analysis (sPD‐1, AUC 0.797; sPD‐L1, AUC 0.760; sCTLA‐4, AUC 0.846; Figure 2A–C).

**TABLE 1:** Target cases of ATL patients and carriers.

Clinical stage		Number of cases (*n*)	
Indolent	Smoldering type	12	14
	Favorable chronic type	2	
Aggressive	Lymphoma type	18	78
	Acute type	49	
	Unfavorable chronic type	11	
Carriers		4	4

**TABLE 2:** Patient background and measurement data.

	Whole patients	Aggressive patients	Indolent patients	Carriers
	(*n* = 96)	(*n* = 78)	(*n* = 14)	(*n* = 4)
	Median (25%–75%)	Median (25%–75%)	Median (25%–75%)	Median (25%–75%)
Age (years)	65 (57–72)	66 (59–72)	66 (54–73)	45 (27–55)
ATL cell (%)	7.8 (0.5–35.8)	7.5 (0.5–42.6)	5.5 (0.9–17.5)	0.3 (0–1.3)
sIL‐2R (U/mL)	3451 (1100–12,550)	5430 (1441–20,575)	943 (553–1713)	440 (278–1033)
sPD‐1 (pg/mL)	454 (198–1448)	697 (242–2067)	206 (129–369)	155 (130–219)
sPD‐L1 (pg/mL)	300 (214–433)	315 (230–501)	200 (165–388)	190 (178–250)
sCTLA‐4 (pg/mL)	10.1 (3.2–36.8)	13.6 (4.2–70.0)	3.0 (1.8–5.3)	2.0 (1.5–2.3)

**FIGURE 3:**
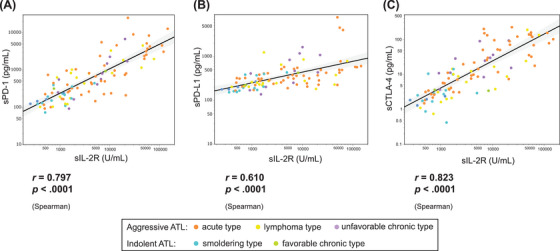
Correlation between immune checkpoint markers and soluble IL‐2R. (A) sPD‐1, (B) sPD‐L1, (C) sCTLA‐4. Correlation coefficients were calculated by Spearman's rank correlation.

There was a significant correlation between these markers and sIL‐2R (sPD‐1, *r* = 0.80, *p* < 0.001; sPD‐L1, *r* = 0.61, *p* < 0.001; sCTLA‐4, *r* = 0.82, *p* < 0.001; Figure 3A–C). There was also a high correlation between each immune checkpoint molecule (sPD‐1 vs. sPD‐L1, *r* = 0.73, *p* < 0.001; sPD‐1 vs. sCTLA‐4, *r* = 0.74, *p* < 0.001; sPD‐L1 vs. sCTLA‐4, *r* = 0.57 vs. *p* < 0.001; Figure S1).

**FIGURE 4:**
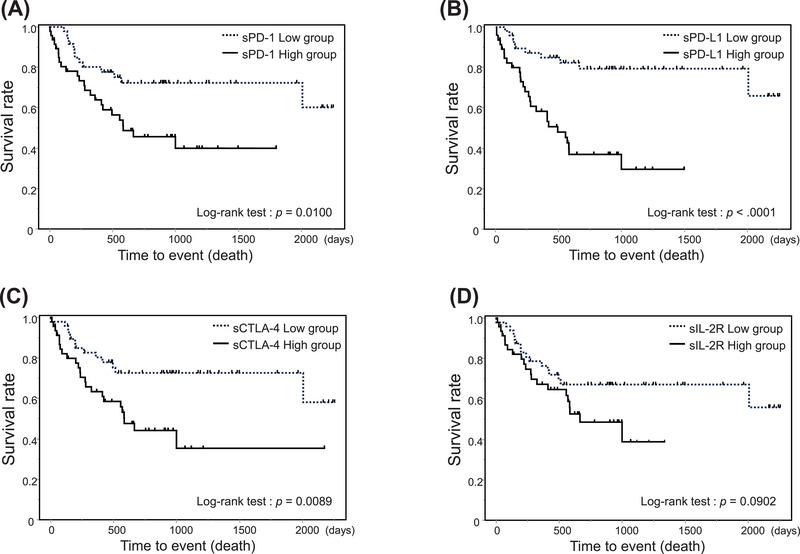
Relationship between the prognosis (death) in ATL and each immune checkpoint molecule. (A) sPD‐1, (B) sPD‐L1, (C) sCTLA‐4, (D) sIL‐2R. Solid line: high‐concentration group, dotted line: low‐concentration group. The high‐ and low‐concentration groups were separated by the median.

After excluding HTLV‐1 carriers, patients were grouped according to the median level of each soluble factor. The probability of 2‐year OS was 72.1% (95% CI, 69.2%–83.6%) in the low sPD‐1 group and 45.7% (95% CI, 31.2%–61.0%) in the high sPD‐1 group (*p* < 0.01; Figure 4A). The probability of 2‐year OS was 79.1% (95% CI, 64.2%–88.9%) in the low sPD‐L1 group and 37.0% (95% CI, 23.5%–52.9%) in the high sPD‐L1 group (*p* < 0.01; Figure 4B). The probability of 2‐year OS was 72.6% (95% CI, 57.5%–83.8%) in the low sCTLA‐4 group and 44.2% (95% CI, 29.5%–59.9%) in the high sCTLA‐4 group (*p* = 0.01; Figure 4C). The probability of 2‐year OS was 67.0% (95% CI, 52.2%–79.1%) in the low sIL‐2R group and 48.5% (95% CI, 32.7%–64.6%) in the high sIL‐2R group (*p* = 0.09; Figure 4D).

In a multivariate analysis for OS, the elevation of soluble ICs was independently associated with inferior OS (sPD‐1, HR 2.42, 95% CI, 1.21–4.84, *p* = 0.01; sPD‐L1, HR 4.38, 95% CI, 2.05–9.35, *p* < 0.01; sCTLA‐4, HR 2.42, 95% CI, 1.22–4.78, *p* = 0.01; Table 3). When the analysis was restricted to aggressive ATL (*n* = 78), sPD‐L1 remained associated with inferior OS (HR 3.83, 95% CI, 1.78–8.26, *p* < 0.01, Table S1a). The results were similar after adjustment for the aggressive ATL prognostic index ATL‐PI (HR 3.60, 95% CI, 1.42–9.17, *p* < 0.01) (Table S1b). Moreover, when the aggressive group was divided into non‐transplant (*n* = 38) and transplant (*n* = 40) groups, sPD‐L1 was a significant hazard factor in both groups (non‐transplant group; HR 4.66, 95% CI, 1.06–20.44, *p* = 0.04; transplant group: HR 3.43, 95% CI, 1.30–9.07, *p* = 0.01; Table S2).

**TABLE 3:** A multivariate Cox regression analysis for overall mortality.

			95% reliability interval		*p*‐value
Variable		Hazard ratio	Under limit	Upper limit	
Age					
	High: > 66 (vs. Low)	2.16	1.12	4.19	0.0224
sIL‐2R					
	High: > 3869 (vs. Low)	1.76	0.91	3.43	0.0944
sPD‐1					
	High: > 510.3 (vs. Low)	2.42	1.21	4.84	0.0126
sPD‐L1					
	High: > 313.0 (vs. Low)	4.38	2.05	9.35	0.0001
sCTLA‐4					
	High: > 10.7 (vs. Low)	2.42	1.22	4.78	0.0112

## 3 Discussion

We assessed the levels of soluble ICs in patients with ATL and found that the levels of these markers were significantly higher in aggressive ATL than in indolent ATL. Furthermore, the level of soluble ICs was a significant prognostic factor in ATL patients.

For the first time, we measured the levels of soluble immune checkpoints (ICs) in patients with ATL and observed elevated levels of these markers, particularly in patients with aggressive ATL. ATL itself was reported to express ICs, and there is also a possibility that these soluble ICs could be secreted from microenvironments [4–6, 23]. Previous reports demonstrated that the expression of immune checkpoint molecules was associated with the adverse clinical outcome in patients with ATL [4, 6]. Therefore, when levels of soluble ICs are elevated, the prognosis of ATL patients could be poor. There was a report that soluble ICs could be associated with the efficacy of IC inhibitors in patients with non‐small cell lung cancer [24]. In ATL patients, it was reported that hyperprogression could occur after the administration of IC inhibitors [25, 26, 27, 28]. Therefore, developing a useful predictive marker for the effectiveness and safety of IC inhibitors in patients with ATL is crucial.

We demonstrated that the level of soluble immune checkpoint molecules was a prognostic factor in patients with ATL. As sIL‐2R is an established biomarker in patients with ATL [29, 30, 31], other soluble forms of ICs could be also meaningful as prognostic markers, considering the strong association between sIL‐2R levels and other soluble ICs. Regarding the soluble factors assessed, their levels were higher in the aggressive type than in the indolent type. However, its significance as a prognostic factor for overall mortality was robust only for sPD‐L1 levels. It is possible that sPD‐L1 levels could be related to differences in the characteristics of ATL cells. The molecular mechanisms contributing to poor clinical outcomes in patients with high sPD‐L1 levels should be clarified in future studies. The number of cases in the current dataset may still be insufficient to establish a new prognostic index that incorporates these markers. There is a possibility that the incorporation of soluble ICs could improve prognostication in patients with ATL. However, as there was a significant correlation among the soluble ICs, it is unclear how many of these soluble ICs should be incorporated in the prognostic index in patients with ATL.

The present study was associated with several limitations. First, the number of cases in our cohort was still limited, which made it difficult to conduct a comparison between the subgroups in four clinical subtypes of ATL. Instead, we used the classification of indolent and aggressive ATL. Thus, our findings should be re‐confirmed in the future in detail using an additional dataset. Second, we did not have data on the expression of these ICs in ATL cells. We were therefore unable to determine the mechanism by which soluble IC levels were elevated in certain patients. For example, the genetic alteration of PD‐L1 was detected in some patients with ATL. This could lead to the elevation of soluble PD‐L1 following the increased expression of PD‐L1 in ATL cells [32]. The mechanisms that lead to the elevation of sPD‐L1 should be clarified in the future. Third, we did not have data on the immune function in each sample. There is a possibility that soluble ICs could affect the immune function, as demonstrated in other fields [7, 10, 11]. However, we were not able to assess the correlation between the level of soluble ICs and the immune function in patients with ATL.

In conclusion, soluble IC levels were significantly higher in aggressive ATL than in indolent ATL, reflecting the disease and immune status of patients with ATL. Although the significant impact of soluble ICs on the clinical outcome was observed in this dataset, the clinical significance of these markers should be investigated in larger studies.

## Author Contributions

S.F. and T.S. designed the research study. J.M., K.T., I.C., H.K., Y.T., Y.S., S.Y., T.Y., and J.I collected data. T.S., C.S., M.K., and Y.I performed the measurement of soluble markers. S.F. and T.S. analyzed the data and wrote the manuscript. All the authors agreed to submit the final version of the manuscript.

## Ethics Statement

Local ethics committee approval was obtained (Osaka International Cancer Institute, No. 23103).

## Patient Consent Statement

All participating patients provided their written informed consent before registration in this study.

## Conflicts of Interest

Sysmex Corporation had the following involvement with the study: conception and design, acquisition of data, analysis and interpretation of data, and review of the manuscript. Four authors, T.S., C.S., M.K., Y.I., collaborated this work as employees of Sysmex Corporation, and managed the contract research for this project. Other authors declare no conflicts of interest in association with the present study.

## Supporting information



Supporting Information

Supporting Information

## Data Availability

The data of this study are not publicly available due to ethical restrictions stipulating that providing such data would exceed the scope of patients’ consent for research use.
