# Supplementary material for: Clinical Significance of Elevated Levels of Soluble‐Form Immune Checkpoint Molecules in Patients With Aggressive Adult T‐Cell Leukemia‐Lymphoma
Source: EJHaem. 2025 Jun 26;6(4):e70046. doi: 10.1002/jha2.70046 (PMC12199990; doi:10.1002/jha2.70046)
Supplement: Supplementary file 1 — Supporting Information [file JHA2-6-e70046-s002.pdf]

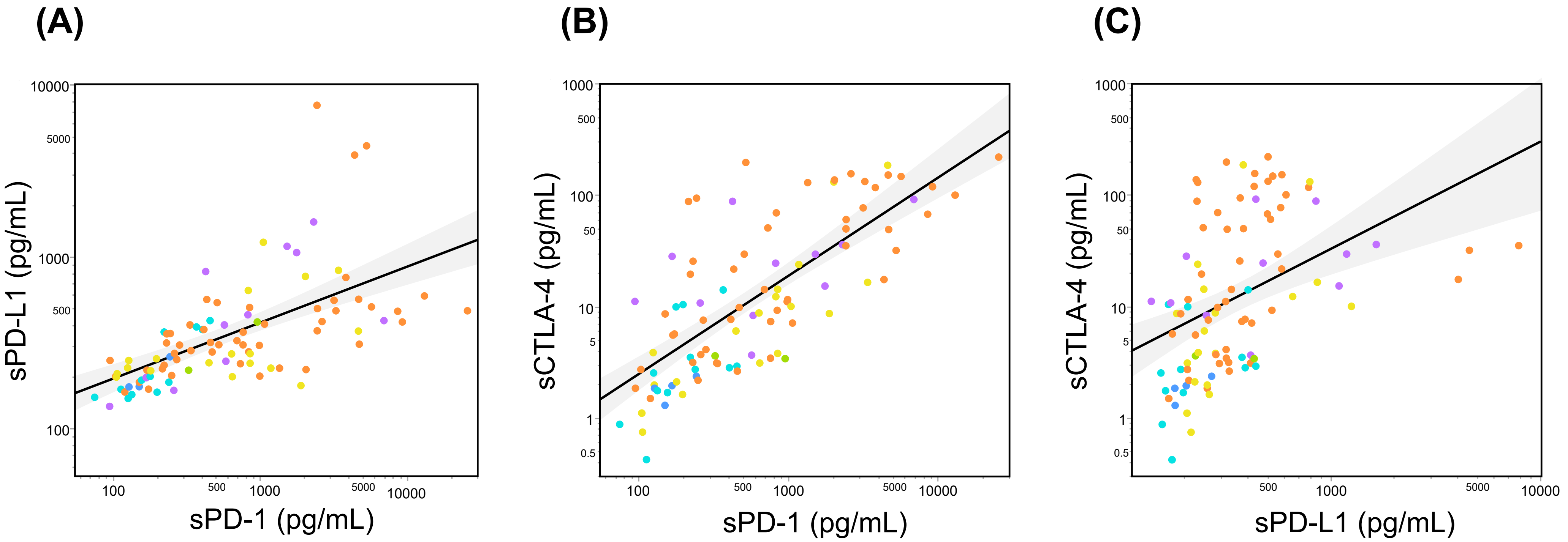

***r* = 0.731**
***p* < .0001**

(Spearman)

***r* = 0.744**
***p* < .0001**

(Spearman)

***r* = 0.567**
***p* < .0001**

(Spearman)

Aggressive ATL: acute type, lymphoma type, unfavorable chronic type

Indolent ATL: smoldering type, favorable chronic type
