# Supplementary material for: Clinical Significance of Elevated Levels of Soluble‐Form Immune Checkpoint Molecules in Patients With Aggressive Adult T‐Cell Leukemia‐Lymphoma
Source: EJHaem. 2025 Jun 26;6(4):e70046. doi: 10.1002/jha2.70046 (PMC12199990; doi:10.1002/jha2.70046)
Supplement: Supplementary file 2 — Supporting Information [file JHA2-6-e70046-s001.docx]

**Supplementary materials**


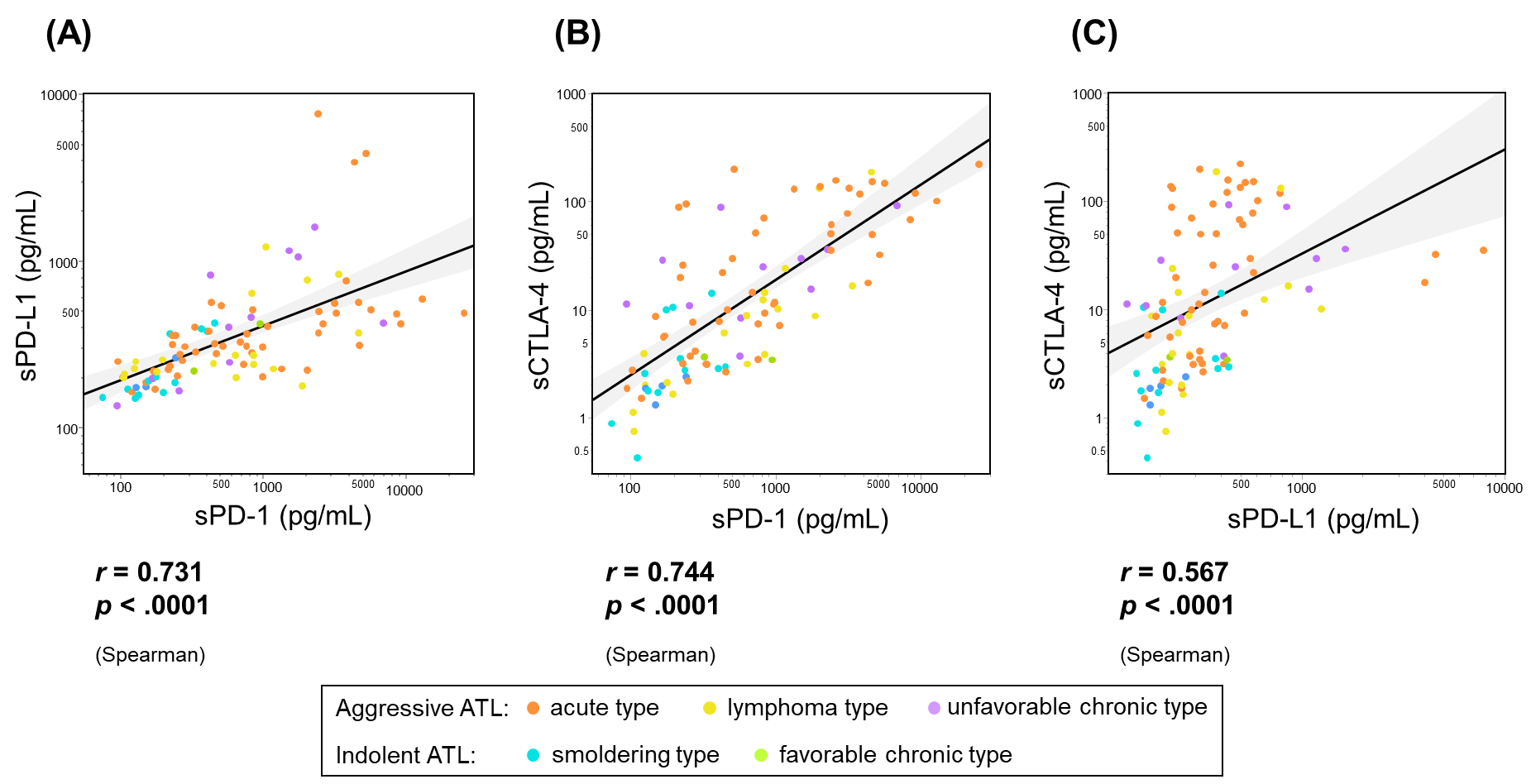


Supplemental Figure 1. Correlation among immune checkpoint markers

1. sPD-1 vs. sPD-L1, (B) sPD-1 vs. sCTLA-4, (C) sPD-L1 vs. sCTLA-4

Supplemental Table 1. A multivariate Cox regression analysis (only aggressive ATL: n=78)

1. No adjustment

| Variable | | Hazard ratio | 95% reliability interval | | *p*-value |
| --- | --- | --- | --- | --- | --- |
|  |  |  | under limit | upper limit |  |
| Age | |  |  |  |  |
|  | High: > 66 (vs. Low) | 2.21 | 1.12 | 4.35 | 0.0223 |
| sIL-2R | |  |  |  |  |
|  | High: > 3869 (vs. Low) | 1.38 | 0.70 | 2.75 | 0.3566 |
| sPD-1 | |  |  |  |  |
|  | High: > 510.3 (vs. Low) | 1.87 | 0.91 | 3.84 | 0.0883 |
| sPD-L1 | |  |  |  |  |
|  | High: > 313.0 (vs. Low) | 3.83 | 1.78 | 8.26 | 0.0006 |
| sCTLA-4 | |  |  |  |  |
|  | High: > 10.7 (vs. Low) | 1.86 | 0.92 | 3.77 | 0.0841 |

1. Adjustment for ATL-PI index

| Variable | | Hazard ratio | 95% reliability interval | | *p*-value |
| --- | --- | --- | --- | --- | --- |
|  |  |  | under limit | upper limit |  |
| Age | |  |  |  |  |
|  | High: > 66 (vs. Low) | 1.69 | 0.81 | 3.54 | 0.1655 |
| sIL-2R | |  |  |  |  |
|  | High: > 3869 (vs. Low) | 1.06 | 0.48 | 2.33 | 0.8854 |
| sPD-1 | |  |  |  |  |
|  | High: > 510.3 (vs. Low) | 1.46 | 0.62 | 3.46 | 0.3865 |
| sPD-L1 | |  |  |  |  |
|  | High: > 313.0 (vs. Low) | 3.60 | 1.42 | 9.17 | 0.0071 |
| sCTLA-4 | |  |  |  |  |
|  | High: > 10.7 (vs. Low) | 1.63 | 0.73 | 3.63 | 0.2326 |

Supplemental Table 2. A multivariate Cox regression analysis (non-transplant and transplant group)

|  | | non-transplant group (aggressive type: n=38) | | | |  | transplant group (aggressive type: n=40) | | | |
| --- | --- | --- | --- | --- | --- | --- | --- | --- | --- | --- |
| Variable | | Hazard ratio | 95% reliability interval | | *p*-value |  | Hazard ratio | 95% reliability interval | | *p*-value |
|  |  |  | under limit | upper limit |  |  |  | under limit | upper limit |  |
| Age | |  |  |  |  |  |  |  |  |  |
|  | High: > 66 (vs. Low) | 1.45 | 0.41 | 5.05 | 0.5615 |  | 4.93 | 1.58 | 15.37 | 0.0060 |
| sIL-2R | |  |  |  |  |  |  |  |  |  |
|  | High: > 3869 (vs. Low) | 4.08 | 0.91 | 18.27 | 0.0658 |  | 0.56 | 0.20 | 1.59 | 0.2731 |
| sPD-1 | |  |  |  |  |  |  |  |  |  |
|  | High: > 510.3 (vs. Low) | 6.61 | 0.87 | 49.94 | 0.0673 |  | 0.92 | 0.35 | 2.42 | 0.8627 |
| sPD-L1 | |  |  |  |  |  |  |  |  |  |
|  | High: > 313.0 (vs. Low) | 4.66 | 1.06 | 20.44 | 0.0413 |  | 3.43 | 1.30 | 9.07 | 0.0130 |
| sCTLA-4 | |  |  |  |  |  |  |  |  |  |
|  | High: > 10.7 (vs. Low) | 9.03 | 1.17 | 69.46 | 0.0344 |  | 0.83 | 0.32 | 2.16 | 0.7080 |
